# Transcranial Direct Current Stimulation Is Safe and Reduces Chronic Fatigue in Patients With Stable Systemic Lupus Erythematosus and Rheumatoid Arthritis

**DOI:** 10.7759/cureus.51462

**Published:** 2024-01-01

**Authors:** Vanessa P De Andrade, Alexandre M Dos Santos, Samuel K Shinjo

**Affiliations:** 1 Rheumatology, Faculdade de Medicina, Universidade de Sao Paulo (FMUSP), São Paulo, BRA

**Keywords:** systemic lupus erythematosus, rheumatoid arthritis, neuromodulation, fatigue disorder, aerobic exercise

## Abstract

To the best of our knowledge, this is the first case series to assess a combined technique of transcranial direct current stimulation (tDCS - a non-pharmacological and non-invasive brain stimulation) and aerobic exercise in one patient with systemic lupus erythematosus (SLE) and another with rheumatoid arthritis (RA) and significant chronic fatigue. We conducted five sessions of tDCS combined with low-intensity treadmill exercise. Fatigue levels were assessed using the Fatigue Severity Scale and the Visual Analog Scale for fatigue before (pre), immediately after five tDCS sessions (post-zero), and after six months (post-6-mo). The level of fatigue decreased, and functionality improved significantly post-zero and remained sustainable post-6-mo in both SLE and RA cases. There was only one mild and transient side effect (headache) specifically in the patient with RA, and no disease reactivation occurred in any of the cases. Our data showed that tDCS combined with aerobic exercise appears to be safe and promising for reducing fatigue and improving functionality in patients with SLE and RA. Randomized studies with larger sample sizes are required to corroborate our findings.

## Introduction

Non-invasive transcranial direct current stimulation (tDCS) is a neuromodulation technique that has emerged in recent years for the treatment of fatigue, such as in patients with fibromyalgia [1] and Sjögren's syndrome [2], and to enhance physical functionality in patients with systemic autoimmune myopathies [3]. Furthermore, these studies have demonstrated that tDCS is safe and does not promote the reactivation of these diseases [2,3]. Other studies on fibromyalgia have suggested that the combination of tDCS and aerobic exercise is more effective in improving fatigue than tDCS alone [4]. Fatigue is a highly prevalent and disabling symptom in patients with systemic autoimmune diseases that negatively affects their quality of life [5].

To the best of our knowledge, no study has yet assessed the impact of tDCS on fatigue in patients with systemic lupus erythematosus (SLE) and rheumatoid arthritis (RA), which motivated the conduct of the present case series. We innovatively evaluated these patients' simultaneous use of tDCS and physical exercise. The safety of this technique in disease reactivation has also been assessed.

## Case presentation

Two patients underwent five sessions of tDCS (2 mA, density of 0.057 mA/cm^2^, anode at C3, and cathode at Fp2, following the International 10/20 System for primary motor cortex localization). The sessions were conducted in the morning at the same time, with one session per day for five consecutive days. Each session lasted for 20 min, with a 10-second ramp-up and 10-second ramp-down. Simultaneously with tDCS, the patients engaged in a 30-minute aerobic exercise on a treadmill at light intensity (Borg scale [6] up to three), including five minutes of warm-up and five minutes of cool-down.

The patients were assessed at three-time points: (a) the week before the tDCS + aerobic exercise application (pre), (b) one week after the completion of the protocol (post-zero), and (c) six months after the completion of the protocol (post-6-mo).

Fatigue levels were assessed using the Fatigue Severity Scale (FSS; range: 9-63; severe fatigue: 36) [7] and the Visual Analog Scale (VAS; range: 0-10) for fatigue. Central sensitization was also evaluated using the Central Sensitization Inventory [8] (with a score ≥25 points indicating sensitization). Activities of daily living were assessed using the Health Assessment Quality (HAQ; range: 0.00-3.00) [9], and the level of physical activity was assessed using the International Physical Activity Questionnaire (IPAQ) [10].

Case 1

A 52-year-old female patient diagnosed with SLE at the age of 33 years presented with mucocutaneous and articular involvement, photosensitivity, digital vasculitis, and a speckled antinuclear antibody pattern of 1/640 (American College of Rheumatology / European League Against Rheumatism [ACR/EULAR] classification criteria for SLE) [11]. She had been using hydroxychloroquine sulfate (5 mg/kg/day) exclusively and had been in remission for 10 years, with an SLE Disease Activity Index 2000 (SLEDAI-2K) of zero. However, she had been experiencing fatigue and diffuse myalgia for eight months. The patient did not have an associated diagnosis of fibromyalgia but was using amitriptyline chronically for insomnia.

**Figure 1:**
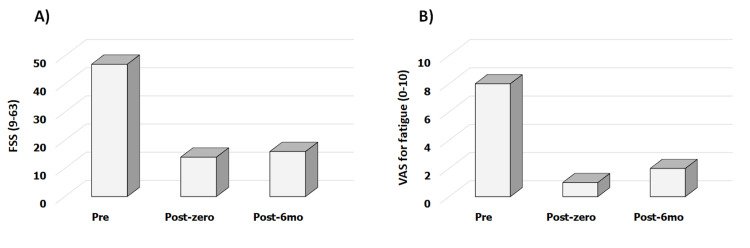
Fatigue Severity Scale (FSS) and Visual Analog Scale (VAS) for fatigue in systemic lupus erythematosus pre - the week before the tDCS and aerobic exercise application; post-zero - one week after the completion of the protocol; post-6-mo - six months after the completion of the protocol tDCS - transcranial direct current stimulation

In the pre-tDCS session assessment, the patient had an FSS score of 47 (Figure 1A) and VAS for fatigue of eight (Figure 1B). The patient had a central sensitization scale score of 59 points and HAQ score of 0.63 and was physically inactive.

After five sessions of the protocol, the FSS decreased by 70.2% (14 points), the central sensitization scale decreased by 25.0% (44 points), VAS for fatigue decreased by 87.5% (1.0), and HAQ decreased by 60.0% (0.25). All these parameters were sustained for six months after follow-up. The physical activity levels remained unchanged throughout the study period.

Throughout the course of the protocol, there was no disease reactivation or medication changes. As the only adverse effect of the technique, the patient experienced mild transient headaches during the initial days of the protocol administration.

Case 2

A 36-year-old female patient diagnosed with RA at the age of 20 years presented with polyarthritis affecting both small and large joints, positive rheumatoid factor, and no extra-articular manifestations (ACR/EULAR classification criteria for RA) [12]. The patient had been receiving tocilizumab (8 mg/kg intravenously, every four weeks) for six years in combination with leflunomide (20 mg/day), and her disease was in remission with a disease activity score (DAS28) of 2.4. The patient did not have fibromyalgia, a history of psychiatric comorbidities, or central medication use. However, she experienced significant diffuse fatigue and myalgia for more than six months.

**Figure 2:**
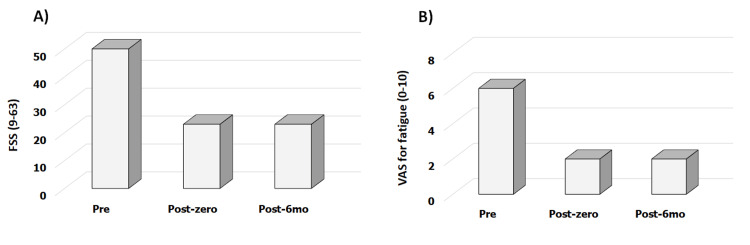
Fatigue Severity Scale (FSS) and Visual Analog Scale (VAS) for fatigue in rheumatoid arthritis pre - the week before the tDCS and aerobic exercise application; post-zero - one week after the completion of the protocol; post-6-mo - six months after the completion of the protocol tDCS - transcranial direct current stimulation

In the pre-tDCS session assessment, the patient had an FSS score of 53 (Figure 2A) and VAS for fatigue of 6.0 (Figure 2B). Moreover, the patient had a central sensitization scale score of 48 points, HAQ score of 0.75, and was physically inactive.

After five sessions of the protocol, FSS decreased by 57.0% (23 points), the central sensitization scale decreased by 54.0% (22 points), VAS for fatigue decreased by 66.7% (2.0), and HAQ decreased by 67.0% (0.25). All these parameters were sustained for six months after follow-up. The physical activity levels remained unchanged throughout the study period.

Throughout the course of the protocol, there was no disease reactivation or medication changes. In addition, the patient did not experience any significant adverse effects from tDCS.

## Discussion

To the best of our knowledge, this is the first case series to demonstrate that tDCS combined with aerobic exercise effectively reduced fatigue in patients with autoimmune rheumatic diseases (SLE and RA) and that this reduction was sustained over time. Furthermore, the technique was found to be safe, with no evidence of disease reactivation.

While the assessment involved only two patients without a control group, this is indeed groundbreaking. Moreover, including patients with well-defined diagnoses based on the ACR/EULAR classification criteria [11,12] for their respective autoimmune diseases adds robustness. The selection of two patients without a history of fibromyalgia or psychiatric conditions that could confound the analysis of the results is noteworthy. Finally, the extended follow-up period enhanced the comprehensive evaluation of this assessment.

Our patients exhibited a high degree of central sensitization, which may have contributed to the elevated fatigue levels observed. Although it is well established in the literature that fibromyalgia is a prototype of central sensitization syndrome, we found evidence of this process in systemic autoimmune rheumatic diseases, regardless of the presence of associated fibromyalgia [13-15]. Furthermore, central sensitization is associated with fatigue in rheumatologic diseases, irrespective of the presence of musculoskeletal symptoms and disease activity [14,15].

Thus, we can conclude that fatigue in systemic autoimmune rheumatic diseases has a central component in addition to a peripheral component. It is precisely in this central component that the justification for neuromodulation lies, as it encompasses processes capable of modifying abnormal patterns of functioning, relying on cerebral neuroplasticity primarily through creating and activating new neural networks [15].

In our patients, there was a decrease of at least 25% in the Central Sensitization Inventory scores [10], possibly due to a synergistic mechanism resulting from the combination of a peripheral stimulus (aerobic exercise) and a central stimulus (neuromodulation). This combination may disrupt potential mechanisms that contribute to central-level fatigue, particularly through neuroplastic changes.

Seeking safety in the protocol, given the rarity of autoimmune diseases that may undergo reactivation due to environmental triggers, we conducted five sessions of tDCS combined with aerobic exercise. The number of sessions was both safe and effective.

As a highlight of our analysis, we assessed the long-term effects of the therapy (six months after the intervention). Additionally, we observed that the physical activity levels of the patients did not change and that there were no changes in their medication therapy. These factors could be potential biases for sustained improvement of fatigue in these patients.

It is noteworthy that there was an improvement in the HAQ [11], extending the improvement from fatigue to enhanced functionality and consequently improving the quality of life of the patients.

Therefore, the present case series demonstrated that tDCS sessions combined with aerobic exercise are safe and effective for sustaining fatigue reduction in patients with RA and SLE. Further randomized studies with larger sample sizes are required to confirm our results.

## Conclusions

tDCS is a non-invasive neuromodulation technique used to treat fatigue. Fatigue is a highly prevalent and disabling symptom in patients with rheumatic autoimmune diseases that negatively affects their quality of life. Our case series showed that tDCS sessions combined with aerobic exercise effectively reduced fatigue in RA and SLE. Furthermore, the technique was found to be safe, with no evidence of disease reactivation.
